# Are medication effects on subjective response to alcohol and cue-induced craving associated? A meta regression study

**DOI:** 10.1007/s00213-023-06409-4

**Published:** 2023-07-15

**Authors:** Lara A. Ray, Steven J. Nieto, Lindsay R. Meredith, Elizabeth Burnette, Suzanna Donato, Molly Magill, Han Du

**Affiliations:** 1 Department of Psychology, University of California at Los Angeles, 1285 Franz Hall, Box 951563, Los Angeles, CA 90095-1563 USA; 2 Department of Psychiatry and Biobehavioral Sciences, University of California at Los Angeles, Los Angeles, CA USA; 3 Brain Research Institute, University of California at Los Angeles, Los Angeles, CA USA; 4 Center for Alcohol and Addiction Studies, Brown University School of Public Health, Providence, RI USA

**Keywords:** Alcohol use disorder, Pharmacotherapy, Alcohol administration, Cue-reactivity, Sedation, Alcohol craving

## Abstract

**Rationale:**

Alcohol administration and cue-reactivity paradigms are frequently used to screen for the initial efficacy of medications for alcohol use disorder (AUD). While medication effects on the primary outcomes for these paradigms are assumed to be qualitatively related, there is a critical lack of quantitative evidence to support this hypothesis.

**Objectives:**

The study aims to test the relationship between medication effect sizes on subjective response to alcohol administration and medication effect sizes for cue-induced craving to cue exposure, using meta-analysis.

**Methods:**

Systematic literature searches were conducted to identify randomized trials, wherein AUD medications were tested using the alcohol administration and/or cue-reactivity paradigms. From these studies, descriptive statistics were collected to compute medication effect sizes on the primary outcomes for each respective paradigm. With medication as the unit of analysis, medication effect sizes in alcohol administration studies were compared with medication effect sizes in cue-reactivity studies using the Williamson-York regression which allows for meta-regression across independent samples.

**Results:**

Medication effect sizes on alcohol-induced stimulation and alcohol-induced craving were not significantly associated with medication effect sizes on cue-induced alcohol craving (*k* stimulation = 10 medications, $\widehat{\beta }=0.272 \left(\mathrm{SE}=0.263\right), p=0.150$ and *k* craving = 11 medications, $\widehat{\beta }=0.217$ (SE = 0.237), $p=0.181$), respectively. Medication effect sizes on alcohol-induced sedation were significantly associated with medication effects on cue-induced craving (*k* = 10 medications, $\widehat{\beta }=-0.600$ (SE = 0.258), $p=0.010$), such that medications that increased alcohol-induced sedation were more likely to reduce cue-induced alcohol craving.

**Conclusions:**

With the exception of alcohol-induced sedation, there is little quantitative evidence of medication effects on subjective response domains measured during alcohol administration parallel medication effects on cue-induced alcohol craving. To provide additional context to the current study, future work should examine whether cue-reactivity findings predict clinical trial outcomes.

**Supplementary Information:**

The online version contains supplementary material available at 10.1007/s00213-023-06409-4.

## Introduction 

Behavioral pharmacology has a long and rich history in addiction science, from examining alcohol effects under controlled laboratory conditions to testing risk mechanisms for alcohol and other substance use disorders (Miranda et al. 2019; Pickens 1977). Behavioral pharmacology approaches have been proposed as tools for medication development for alcohol use disorder (AUD) (Comer et al. 2008; Litten et al. 2012, 2020; Yardley and Ray 2017), with the most commonly used paradigms consisting of controlled alcohol administration (i.e., alcohol “challenge”) and alcohol cue presentation (i.e., alcohol “cue exposure”).

In early phase clinical trials, controlled alcohol challenges allow for tests of medication × drug/alcohol interactions, which is critical in medication development for establishing safety and tolerability. Beyond safety, behavioral pharmacology paradigms permit testing of theoretically meaningful endpoints, described as early efficacy markers, using experimental methods designed to provoke an acute response. These endpoints often include medication-induced changes in the subjective response (SR) to alcohol, such as stimulation and sedation, as well as measures of craving, via cue-reactivity or alcohol/drug administration (Metz et al. 2017; Ray et al. 2015, 2017). Studies of alcohol in the human laboratory often include craving measures in addition to subjective response measures while other studies focus on cue-reactivity as a related and often parallel measure of early efficacy (Miranda et al. 2008; Ray et al. 2011). Individuals who experience the stimulant effects of alcohol and are less sensitive to the sedative effects of alcohol report higher levels of craving, which is thought to indicate greater reward sensitivity and associated “wanting” of alcohol (King et al. 2021, 2016). To the degree to which alcohol cue-reactivity is a measure of wanting of alcohol, we expect that higher stimulation and lower sedation would be associated with greater wanting/craving during cue exposure. Likewise, cue-induced craving and alcohol-induced craving should be associated both conceptually and phenomenologically.

In medication development for AUD, it is argued that these early efficacy endpoints can inform clinical trials and whether or not a novel compound should be advanced to the next stage of clinical testing (i.e., randomized clinical trial) (Litten et al. 2014, 2020). While the utility of behavioral pharmacology for establishing the safety and tolerability of addiction pharmacotherapies in humans is well established, the degree to which the early efficacy of novel compounds in the human laboratory can predict clinical efficacy remains unclear.

In a recent critique, we argued that the degree to which a behavioral pharmacology paradigm is useful as an early efficacy marker depends on the degree to which that paradigm is related to the desired clinical outcome (e.g., abstinence or reduced heavy drinking) (Ray et al. 2018). At a theorical level, medications that reduce alcohol use in real-world settings are expected to reduce alcohol-induced stimulation and craving (Ray et al. 2010a), potentiate the sedative effects of alcohol (Ray et al. 2010a), and reduce cue-induced craving for alcohol in the laboratory (Miranda et al. 2020b). Nevertheless, the association across these putative mechanisms of action of AUD pharmacotherapies has not been empirically tested and doing so is the focus of the present study. In our previous work, we conducted a series of Monte Carlo simulations to determine the required sample size for a behavioral pharmacology study to detect early medication efficacy, based on varying degrees of correlation between human laboratory paradigms outcomes and clinical outcomes (Ray et al. 2018). These simulations used hypothetical parameter values, given that the true association between medication effects in behavioral pharmacology studies and clinical efficacy in randomized clinical trials (RCTs) remain unknown. Our laboratory then implemented a novel meta-analytic method to examine whether human laboratory tests of medication effects predict medication outcomes in RCTs (Ray et al. 2021). We searched the literature for AUD medications tested in both the human lab and in RCTs. For the human lab studies, we computed medication effects on stimulation, sedation, and craving during alcohol administration (*k* = 51 studies, 24 medications). For RCTs, we computed medication effects on abstinence and heavy drinking (*k* = 118 studies, 17 medications). Results revealed that medications that reduced stimulation, increased sedation, and reduced craving during alcohol administration were associated with better clinical outcomes, supporting the predictive utility of behavioral pharmacology outcomes.

This proof-of-concept study (Ray et al. 2021) demonstrated that human laboratory endpoints track medication effects from the human lab to clinical trials. Nevertheless, other human laboratory models such as alcohol cue-reactivity have yet to be examined using this approach. Moreover, the relationship between medication effects across subjective response endpoints (i.e., stimulation, sedation, and craving) and alcohol cue-reactivity (i.e., cue-induced craving) remains unknown. The present study addresses this gap by conducting a systematic search and coding of AUD medications tested in the human laboratory using the alcohol cue-reactivity paradigm. This effort resulted in a range of studies across AUD pharmacotherapies (*k* = 36 studies, 16 medications). Given that our recent work examined the effects of AUD medications on stimulation, sedation, and craving during an alcohol administration (Ray et al. 2021), we could then compare these medication effective sizes with those on cue-induced alcohol craving following cue exposure. The goal of the present study is to integrate these two human laboratory methods. To integrate these two sets of independent effect sizes from alcohol administration studies and cue-reactivity studies, we used a novel meta-analytic approach whereby medication (i.e., rather than study) was the unit of analysis. This enabled testing of the association between subjective response to alcohol and cue-induced craving across various AUD pharmacotherapies.

## Methods

### Literature review — alcohol challenge studies

The literature search strategy for alcohol challenge studies has been previously reported in detail in Ray et al. (2021). Key features of the search strategy are provided below:

Inclusion criteria for the alcohol challenge studies were (1) the administration of a pharmacological agent approved or being developed for the treatment of AUD, (2) alcohol administered in the laboratory to a target breath alcohol concentration (BrAC) via alcohol challenge or priming for self-administration, (3) subjective response outcomes measured via self-report questionnaires, (4) reported in the English language or translated to English, and (5) publication in a PubMed indexed journal. Databases were searched through July 2018 and collected data were analyzed through February 1, 2023.

A total of 40 medications were identified by screening peer-reviewed literature reviews on medication approaches to treat AUD. PubMed searches were conducted using search and medical subject heading terms: “alcohol challenge,” “alcohol response,” “response* to alcohol,” “alcohol response,” “alcohol priming,” “alcohol intoxication,” “ethanol intoxication,” “response* to ethanol,” and “ethanol response.”

PubMed searches yielded 1206 studies, 1139 of which were excluded during abstract screening. Sixty-seven were eligible for full-text review, 16 of which were excluded after full-text review. A final sample of 51 alcohol challenge studies were included in the present study. The following subjective response domains were identified as primary outcomes in alcohol challenge studies: stimulation, sedation, and alcohol craving.

### Literature review — cue-reactivity studies

Inclusion criteria for human laboratory alcohol cue exposure trials were as follows: (1) randomized trial including the administration of a pharmacological agent (either approved or being developed for the treatment of AUD) and placebo comparison, (2) alcohol cue exposure paradigm in the laboratory (including during imaging scans), (3) collection of self-reported cue-induced craving, and (4) peer-reviewed articles reported in the English language or translated to English. PubMed searches took place on January 3, 2022, and studies were screened, coded, and analyzed through February 1, 2023. This meta-analysis was not pre-registered.

The literature search strategy was based upon the aforementioned meta-analysis from our laboratory testing the relationship between medication effects on subjective response to alcohol during alcohol administration and clinical RCT endpoints (Ray et al. 2021). PubMed searches were informed by UCLA librarians using search strings on each of the 40 medications with the following search and MeSH terms: “alcohol cue-exposure,” “alcohol cue-reactivity,” “alcohol cue reactivity,” “ethanol craving,” “alcohol craving,” “Cues” (MeSH), and “Craving” (MeSH).

PubMed searches yielded 358 unique studies and 299 studies were excluded at abstract screening. Fifty-nine studies were assessed for eligibility in full-text review with 23 studies excluded at this stage. This resulted in a final sample of 36 cue-reactivity studies across 16 medications that were included in the present study. Cue-induced alcohol craving was the primary endpoint for cue-reactivity studies. For primary craving outcomes for cue-reactivity, authors were contacted regarding data requests for 11 studies with a response rate of 27%.

Screening, extraction, and coding of study outcomes and study-level descriptive information (alcohol challenge and cue-reactivity studies) were conducted by two independent raters. Where coding discrepancies existed, all raters met to reach a consensus. Furthermore, when sufficient data to generate effect size estimates were not reported in the published paper, corresponding authors were contacted via email to obtain the necessary information. DigitizeIt software (Bormann 2012) was utilized when necessary to extract descriptive statistics, such as means and standard error, from published figures (Rakap et al. 2016). While it was not an inclusion requirement that cue-reactivity testing take place under breath alcohol concentration (BrAC) of 0.00 g/dl, all but one study (Ray et al. 2011) conducted cue-reactivity testing after verifying alcohol abstinence. For the study that included a post-placebo and a post-alcohol cue-reactivity condition, we estimated the effect size in the post-placebo cue-exposure condition.

### Data analytic plan

Data analysis for this study consisted of several steps. First, we calculated the unbiased Cohen’s *d* as the target effect size for each alcohol challenge or cue-reactivity study. Cohen’s *d* was calculated for each subjective response outcome and defined as the mean of the active medication group minus the mean of the control group (${\overline{y} }_{\mathrm{medication}}-{\overline{y} }_{\mathrm{control}}$) divided by the pooled standard deviation. Cohen’s *d* was corrected by multiplying a correction factor to obtain an unbiased Cohen’s *d*. For the alcohol challenge studies, there are three outcomes: Stimulation/Hedonia, Craving/Motivation, and Sedation/Motor Intoxication. For the cue-reactivity studies, there is only one outcome: Craving/Motivation. For Stimulation and Craving, the treatment group is expected to have a lower group mean than the control group. Since Cohen’s *d* was computed as (${\overline{y} }_{\mathrm{medication}}-{\overline{y} }_{\mathrm{control}}$), a negative effect size indicates that the treatment group is more effective than the control group. But for Sedation, the treatment group is expected to have a higher group mean than the control group; therefore, a positive effect size indicates that the treatment group is more effective than the control group.

Second, for each medication, we used fixed-effect meta-analysis to compute the averaged effect size with the *metaphor* R package (Viechtbauer 2010). Then, each medication had one averaged effect size within each outcome. Fixed-effect meta-analysis was used instead of random-effect meta-analysis because for some medications, there were only 1 or 2 studies. In this case, we do not have enough studies to accurately estimate both the overall effect size and between-study heterogeneity.

Third, the relationships between medication effect sizes in alcohol challenge studies and medication effect sizes in cue-reactivity studies were examined using the Williamson-York bivariate weighted least squares estimation to preserve the errors in both the independent and dependent variables (Williamson 1968; York 1966; 1968; York et al. 2004). This is useful as it allows for independent samples to be represented at each side of the equation. In other words, independent samples were used to test subjective response outcomes and cue reactivity outcomes. A total of 3 statistical models were used to compare medication effect sizes across cue-reactivity and alcohol challenge studies: (1) medication effect sizes regressed on cue-induced alcohol craving AND medication effect sizes regressed on alcohol-induced stimulation; (2) medication effect sizes regressed on cue-induced alcohol craving AND medication effect sizes regressed on alcohol-induced sedation; and (3) medication effects sizes regressed on cue-induced alcohol craving AND medication effect sizes regressed on alcohol-induced alcohol craving. In these analyses, medication was used as the unit of analysis; therefore, data points for the independent and dependent variables were not derived from the same study (i.e., by design in the current analysis).

## Results

### Cue-induced alcohol craving and alcohol-induced stimulation

**Fig. 1:**
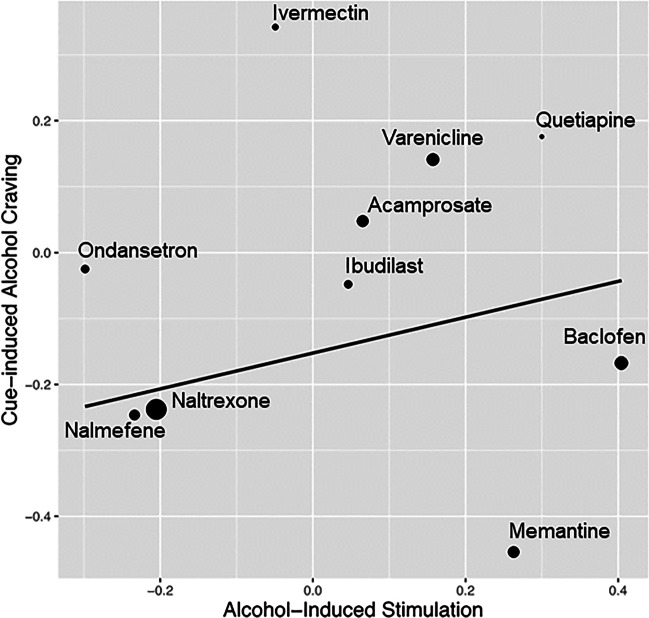
The linear relationship between medication effect sizes on alcohol-induced stimulation and medication effect sizes on cue-induced craving. Each medication is represented as a dot (and labeled) on the regression line and smaller dots indicate more error variance while larger dots indicate less error variance around each estimate

We tested the relationship between medication effect sizes for alcohol-induced stimulation within alcohol challenge studies and those of cue-induced alcohol craving within cue-reactivity studies using the Williamson-York regression. Our aim was to test whether the effect sizes of alcohol-induced stimulation would be correlated with the effect sizes of cue-induced alcohol craving across the two experimental designs. Each dot in Fig. 1 represents a medication, with larger dots indicating smaller sampling errors and greater weights. The *x*-axis shows the magnitude of alcohol-induced stimulation effect sizes and the *y*-axis shows the magnitude of cue-induced alcohol craving effect sizes. A total of 10 AUD medications were represented in Fig. 1. Effect size estimations for cue-induced alcohol craving and alcohol-induced stimulation are presented in Tables 1 and 2, respectively.

**Table 1:** Estimated effect sizes and standard errors for each medication for the outcome of cue-induced alcohol craving

Medication	Number of effect sizes	Estimated effect size (Cohen’s *d*)	Standard error
Baclofen	10	− 0.1674	0.1337
Prazosin	2	− 0.2927	0.2674
Varenicline	4	0.1412	0.1484
Varenicline + naltrexone	1	− 0.4978	0.3665
Acamprosate	4	0.0479	0.1396
Olanzapine	3	− 0.3628	0.1873
Nalmefene	3	− 0.2462	0.1934
Memantine	2	− 0.4541	0.1643
Naltrexone	12	− 0.2377	0.0773
Naltrexone XR	1	− 0.5930	0.3871
Ondansetron	1	− 0.0247	0.2918
Naltrexone + ondansetron	1	− 0.6400	0.3103
Quetiapine	1	0.1759	0.5410
Ibudilast	1	− 0.0480	0.2887
Ivermectin	1	0.3421	0.4295

**Table 2:** Estimated effect sizes and standard errors for each medication for the outcome of alcohol-induced stimulation

Medication	Number of effect sizes	Estimated effect size (Cohen’s *d*)	Standard error
Acamprosate	1	0.0653	0.3181
Aripiprazole	2	− 0.4361	0.1618
Baclofen	2	0.4041	0.1332
Dutasteride	1	− 0.0894	0.1682
Gabapentin	2	0.2154	0.1762
Ibudilast	1	0.0462	0.0947
Idazoxan	1	− 0.6208	0.2764
Ivermectin	1	− 0.0492	0.1477
Mecamylamine	3	− 0.3364	0.0733
Memantine	2	0.2630	0.1770
Nalmefene	1	− 0.23361	0.1744
Naltrexone	16	− 0.2051	0.0245
Ondansetron	2	− 0.2985	0.0864
Quetiapine	1	0.30004	0.2891
Ritanserin	1	− 0.2461	0.1801
Topiramate	1	− 0.4545	0.1861
Varenicline	3	0.1572	0.0837

The estimated slope is $\widehat{\beta }=0.272$ (SE = 0.263, $p=0.150$). The positive relationship suggests that medications that decreased alcohol-induced stimulation in the alcohol challenge studies similarly decreased craving in cue-reactivity studies. We considered a one tailed hypothesis where ${H}_{a}:\beta >0$. In addition, we used Bonferroni correction to adjust for multiple hypothesis tests. In our current study, we conducted three regression models. Hence, the corrected $\alpha$ level is 0.05/3 = 0.017. Compared to the corrected $\alpha$ level, the slope is not significant. Therefore, we fail to claim a positive linear relationship between the effect sizes of alcohol-induced stimulation in the alcohol challenge studies and those in cue-reactivity studies.

### Cue-induced alcohol craving and alcohol-induced sedation

**Fig. 2:**
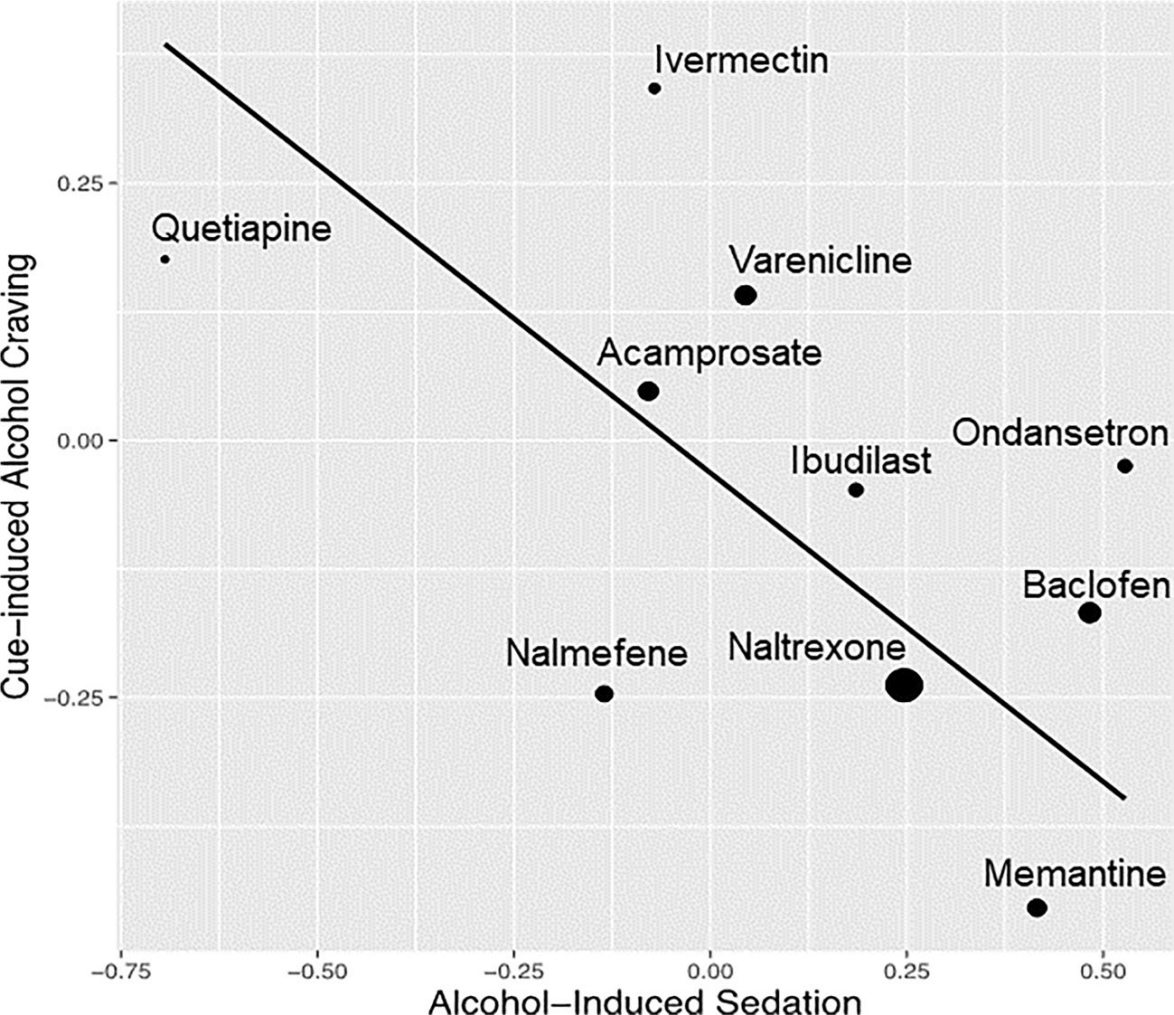
The linear relationship between medication effect sizes on alcohol-induced sedation and medication effect sizes on cue-induced craving. Each medication is represented as a dot (and labeled) on the regression line and smaller dots indicate more error variance while larger dots indicate less error variance around each estimate

We tested the linear relationship between the medication effect sizes of alcohol-induced sedation within alcohol challenge studies and those of cue-induced alcohol craving within cue-reactivity studies. Data was available for 10 medications across both alcohol administration and alcohol cue exposure studies. For the alcohol-induced sedation effect sizes, a positive effect size indicates that the treatment group is more effective than the control group, while for the cue-induced alcohol craving effect sizes, a negative effect size indicates that the treatment group is more effective than the control group. As illustrated in Fig. 2, the estimated slope is $\widehat{\beta }=-0.600$ (SE = 0.258, $p=0.010$). The negative relationship suggests that medications that increase alcohol-induced sedation in the alcohol challenge studies were found to decrease craving in cue-reactivity studies. With the one tailed hypothesis (${H}_{a}:\beta <0$) and the corrected $\alpha$ level of 0.017, the linear slope is statistically significant. Effect size estimations for alcohol-induced sedation are presented in Table 3.

**Table 3:** Estimated effect sizes and standard errors for each medication for the outcome of alcohol-induced sedation

Medication	Number of effect sizes	Estimated effect size (Cohen’s *d*)	Standard error
Acamprosate	2	− 0.0789	0.1806
Aripiprazole	2	0.4147	0.1336
Baclofen	2	0.4826	0.1372
Dutasteride	1	0.3884	0.0703
Finasteride	1	− 0.4793	0.0979
Gabapentin	1	0.2603	0.2032
Ibudilast	1	0.1852	0.1173
Idazoxan	1	0.2712	0.2005
Isoflavone	1	0.0353	0.2282
Ivermectin	1	− 0.0713	0.1795
Mecamylamine	2	0.2333	0.1014
Memantine	1	0.4154	0.1802
Nalmefene	1	− 0.1351	0.1741
Naltrexone	17	0.2462	0.0221
Ondansetron	2	0.5274	0.1367
Quetiapine	1	− 0.694	0.2600
Ritanserin	1	− 0.1189	0.3101
Topiramate	1	0.5591	0.2645
Varenicline	3	0.0445	0.1279
Zonisamide	1	− 0.5925	0.3238

### Cue-induced alcohol craving and alcohol-induced craving

**Fig. 3:**
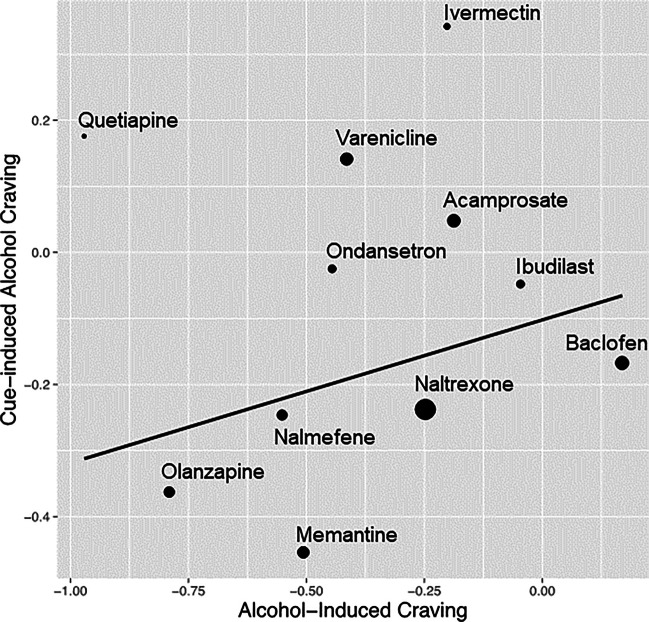
The linear relationship between medication effect sizes on alcohol-induced craving and medication effect sizes on cue-induced craving. Each medication is represented as a dot (and labeled) on the regression line and smaller dots indicate more error variance while larger dots indicate less error variance around each estimate

In the final model, we tested the linear relationship between the medication effect sizes of alcohol-induced craving within alcohol challenge studies and those of cue-induced alcohol craving within cue-reactivity studies. Data was available for 11 medications. Although both sets of effect sizes are for craving outcomes tested in each experimental design, the linear slope is not significant ($\widehat{\beta }=0.217$, SE = 0.237, $p=0.181>0.017$; see Fig. 3). Hence, we fail to conclude that the medications that decreased alcohol-induced craving in the alcohol challenge studies similarly decreased craving in cue-reactivity studies. Effect size estimations for alcohol-induced craving are presented in Table 4.

**Table 4:** Estimated effect sizes and standard errors for each medication for the outcome of alcohol-induced craving

Medication	Number of effect sizes	Estimated effect size (Cohen’s *d*)	Standard error
Acamprosate	2	− 0.1878	0.1549
Aripiprazole	1	0.0412	0.3553
Baclofen	1	0.1687	0.1113
Finasteride	1	− 0.3870	0.1637
Gabapentin	1	− 0.4723	0.2370
Ibudilast	1	− 0.0463	0.0828
Ivermectin	1	− 0.2023	0.1505
Mecamylamine	2	− 0.3781	0.1046
Memantine	2	− 0.5066	0.1263
Nalmefene	1	− 0.5516	0.1769
Naltrexone	15	− 0.2481	0.0329
Olanzapine	2	− 0.7908	0.1557
Ondansetron	2	− 0.4456	0.1495
Quetiapine	1	− 0.9709	0.3088
Topiramate	1	− 0.1189	0.2549
Varenicline	3	− 0.4149	0.1254
Zonisamide	1	− 0.7530	0.3549

To assess for publication bias, we provide funnel plots for all 15 medications for the outcome of cue-reactivity (Supplementary Fig. 1). For most medications, we only have 1 or few effect sizes; hence, it is difficult to reliably assess for publication bias.

## Discussion

There is a great deal of interest in human laboratory paradigms as tools for screening medications for AUD (Litten et al. 2020; Plebani et al. 2012). However, until recently, the vast majority of papers arguing for the utility of human laboratory models in medication development have been qualitative in nature. The present study leverages extensive efforts to systematically search and code for human laboratory studies of alcohol administration and alcohol cue-exposure to test promising pharmacotherapies. Based on our original meta-analysis of pharmacotherapy effects on alcohol administration in the laboratory (Ray et al. 2021), we expanded the search to pharmacotherapy effects on cue-reactivity in the current report. The original focus on subjective response is consistent with its centrality in multiple theories of AUD etiology (Bujarski et al. 2015; King et al. 2011; Ray et al. 2016; Schuckit 1984), and its relevance and wide prevalence in the AUD behavioral pharmacology literature (Litten et al. 2012; Ray et al. 2016, 2010a). The novel methods for integrating and analyzing effect size data from different samples, with different error estimates, has allowed us to continue this line of inquiry. Specifically, given that both the independent and dependent variables have errors, we used the Williamson-York bivariate weighted least squares estimation to preserve the errors in both the independent and dependent variables (Williamson 1968; York 1966, 1968; York et al. 2004). These novel methods allowed us to interrogate the relationship between alcohol administration phenotypes (i.e., alcohol-induced stimulation, sedation, and craving) and the cue-exposure phenotype (i.e., cue-induced craving), using medication as the unit of analysis. This allows us to inform medication development for AUD by examining associations across widely tested early efficacy endpoints. The finding that these endpoints are generally independent of one another argues that comprehensive early efficacy testing including multiple endpoints is warranted.

Results revealed that there was no significant association between cue-induced craving and alcohol-induced craving, such that medications that reduced alcohol-induced craving in the lab were not significantly more likely to reduce cue-induced craving for alcohol during the cue-exposure paradigm. This distinction between cue-induced and alcohol-induced craving is notable. While cue-induced craving may be associated with the likelihood of initiating a drinking episode (i.e., begin drinking), the alcohol-induced craving reports may be most representative of the likelihood of escalating a drinking episode (i.e., heavy drinking). In fact, in our previous studies we modeled cue-reactivity after individuals completed an alcohol challenge and had a breath alcohol concentration of 0.06 g/dl in order to test how the addition of cues to a moderate blood alcohol concentration would be impacted by pharmacotherapy (Ray et al. 2011). Hence, the finding that cue-induced and alcohol-induced craving are dissociable is generally consistent with the literature on human laboratory models and suggests that both phenotypes should be independently tested in pharmacotherapy development for AUD. Further, medications that reduce alcohol-induced craving may decrease heavy drinking as a primary clinical endpoint, whereas medications that reduce alcohol cue-reactivity, on the other hand, may promote abstinence as a primary clinical endpoint.

The findings for alcohol-induced stimulation and cue-induced craving are equally intriguing. There was no significant association between cue-induced craving and alcohol-induced stimulation, such that medications that reduced alcohol-induced stimulation in the lab were not significantly more likely to reduce cue-induced craving for alcohol during the cue-exposure paradigm. This is interesting given that much has been written about medications such as naltrexone, which “block the buzz” from alcohol and how that is part of its mechanisms of action (King et al. 1997; Volpicelli et al. 1995). The effects of naltrexone on subjective responses to alcohol administration, including stimulation and sedation, were verified in a meta-analysis by our laboratory (Ray et al. 2019). Nevertheless, this study comprised 15 medications and while naltrexone is the most widely researched one, the overall findings do not support an association between medication effects on attenuation of alcohol stimulation and same medication effects on cue-induced alcohol craving.

Conversely, a significant relationship was observed between alcohol-induced sedation and cue-induced alcohol craving across the medications studied herein. Specifically, medications that increased alcohol-induced sedation during alcohol administration in the lab were significantly more likely to reduce cue-induced craving for alcohol during the cue-exposure paradigm. Ratings of alcohol-induced sedation are most strongly associated with self-reported ratings of the negative effects of alcohol, measured by the Subjective High Assessment Scale (SHAS) (Ray et al. 2010b). This is highly relevant since a robust body of research, most prominently by Schuckit and colleagues, has demonstrated that high scores on the SHAS are protective against the development of AUD prospectively (King et al. 2016; Schuckit 1984; 1994). Thus, it is plausible that medications that can potentiate the sedative effects of alcohol during alcohol intake are more likely to decrease cue-induced craving for alcohol. As individuals learn that alcohol intake is more aversive, as a result of pharmacotherapy, and as indicated by their ratings of subjective sedation, their craving responses to alcohol cues may become blunted. Related findings from behavioral experiments have shown that the incentive salience of alcohol cues (measured by neurophysiological indices, response bias, and event-related brain potentials) was higher among individuals low in alcohol sensitivity, relative to those with higher alcohol sensitivity (Cofresí et al. 2022; Fleming et al. 2021). Based on these findings, it is plausible that enhancing alcohol sensitivity via pharmacotherapies may decrease the incentive salience of alcohol measured through cue-reactivity.

The study findings, however, do not speak to feelings of sedation that occur outside of the alcohol administration context. While many pharmacotherapies may have sedative properties or side effects, this is not the construct captured in this study. Additionally, given that cue-induced craving for alcohol is associated with negative affect (i.e., feelings of anxiety, “on edge,” and needing to drink) (Ray 2011), it is also plausible that this construct would overlap with the “undesirable” and negative sedative effects of alcohol.

In addition to considering the associations between subjective response outcomes and cue-reactivity, it is useful to review the effect sizes generated over the course of this study. It is notable that combination pharmacotherapy (i.e., varenicline + naltrexone and ondansetron + naltrexone) showed stronger reductions in cue-induced craving, compared to monotherapy. This finding is preliminary given that only one study was available for each combination treatment. Likewise, some medications with some evidence of clinical efficacy, such as varenicline (Litten et al. 2013), do not appear to work by reducing cue-induced alcohol craving according to this meta-analysis. This is consistent with a recent trial specifically focused on the effects of varenicline on cue-reactivity in the laboratory (Miranda et al. 2020a). Naltrexone, on the other hand, shows a small effect size on cue-induced craving which is consistent with previous research (Hendershot et al. 2017) and robust across 12 trials included in this meta-analysis.

There are some important limitations and considerations when interpreting the findings of the current study. First, the study only included medications that were tested in both alcohol administration and alcohol cue-reactivity studies, which may exclude some potentially effective medications not tested using these paradigms. Second, publication bias remains a problem in scientific research and likely influenced medication effect sizes reported in the literature. Third, there was an imbalance in the number of studies available for different medications, which likely affected the precision of the estimates for less studied medications. This imbalance also precludes us from drawing inferences about the specific relationships across subjective response and alcohol cue-reactivity for each of the medications tested and instead focuses on the overall relationship across all medications identified through the systematic search methods.

In closing, this study tested the relationship between alcohol administration phenotypes and the cue-exposure phenotype, using medication as the unit of analysis. Medications that reduced alcohol-induced craving or stimulation using the alcohol administration paradigm were not significantly likely to alter cue-induced alcohol craving during the cue-exposure paradigm. However, medications that increased alcohol-induced sedation were more likely to reduce cue-induced craving. These findings suggest that medication effects on alcohol-induced stimulation and craving do not overlap with medication effects on cue-induced alcohol craving and should be tested separately in pharmacotherapy development for AUD. In clinical practice we must also recognize that medications for AUD may reach clinical efficacy without necessarily reducing alcohol cue-reactivity. Testing of the association between cue-induced craving and clinical trials outcomes represents a next step in establishing the relationship between early efficacy endpoints and clinical efficacy.

## Supplementary Information

Below is the link to the electronic supplementary material.

Supplementary file1 (DOCX 80 KB)
