# Supplementary material for: Are medication effects on subjective response to alcohol and cue-induced craving associated? A meta regression study
Source: Psychopharmacology (Berl). 2023 Jul 15;240(9):1921–30. doi: 10.1007/s00213-023-06409-4 (PMC10471658; doi:10.1007/s00213-023-06409-4)

**Supplementary Figure 1.**

**Supplementary Figure 1.** Funnel plots for all 15 medications for the outcome of cue-reactivity.


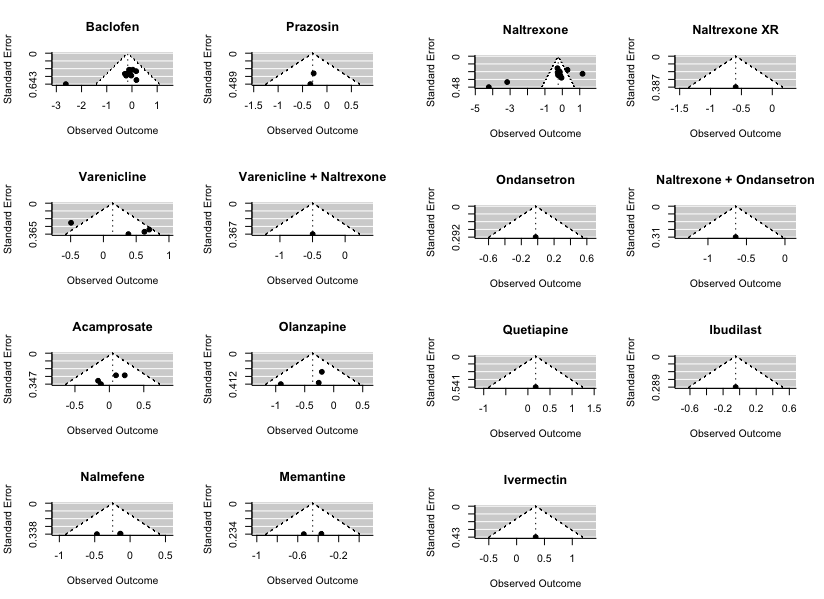

Supplement: Supplementary file 1 — Supplementary file1 (DOCX 80 KB) [file 213_2023_6409_MOESM1_ESM.docx]
